# Bending the epidemic curve: advancements and opportunities to reduce the threat of emerging pathogens

**DOI:** 10.1017/S095026881900058X

**Published:** 2019-04-03

**Authors:** Angel N. Desai, Lawrence C. Madoff

**Affiliations:** 1 Department of Infectious Disease, Brigham and Women's Hospital, Boston, USA; 2 International Society for Infectious Disease, Brookline, USA; 3 University of Massachusetts Medical School, Worcester, USA

**Keywords:** Epidemics, outbreaks

## Abstract

This invited editorial introduces a special issue of *Epidemiology & Infection* while also discussing advances in emerging infectious diseases.

The *World Health Organization* (WHO) recently enumerated 10 threats to global health for 2019, notably emphasising Ebola and other high-threat emerging pathogens as growing priorities [1]. Although many of these diseases have the potential to cause public health emergencies, a lack of timely surveillance and effective interventions continue to hamper preparedness efforts [2]. Moreover, the *annualised* financial impact of a global pandemic has been estimated to be as high as US$80 billion, severely burdening already constrained national budgets and healthcare systems [3]. Outbreaks in resource-limited settings are often further complicated by conflict, fragile health systems, disruptions in healthcare delivery and socio-economic disparities [4]. These factors can make implementing appropriate outbreak prevention and control strategies difficult. To address these shortcomings, investments in surveillance to inform disease forecasting and ultimately effective prevention strategies are paramount to tackling the challenges posed by emerging pathogens.

This edition of *Epidemiology & Infection* highlights new insights into many of the emerging infectious diseases mentioned in the WHO report, as well as re-emerging diseases that are gaining global prominence. They address a variety of diagnostic, therapeutic and epidemiological advances in outbreak preparedness. Several papers review data on priority pathogens with a focus on resource-limited settings. For example, Sikkema *et al*. present a systematic review on Middle East Respiratory Syndrome Coronavirus with the aim of characterising the distribution and spread of infection in dromedary camels [5]. Aditi *et al.* review the recent Nipah virus outbreaks in Bangladesh and India, shedding light on transmission patterns of this emerging pathogen while also highlighting the importance of ongoing surveillance [6]. A review analysis of H5N1 and H9N2 by Parvin *et al*. discusses genetic variations among avian influenza viruses circulating in Bangladesh and the impact of accumulating mutations noted in poultry. Lessons learned from the WHO response to the recent 2017 pneumonic plague outbreak in Madagascar are presented by Heitzinger *et al.*, who highlight specifically the challenges of implementing rapid infection prevention and control measures in epidemic settings [7]. These studies also underscore the critical importance of the One Health approach [8]. These, and indeed the great majority of emerging disease threats, are zoonotic and require us to consider other hosts and the environment in addressing them.

Outbreaks of re-emerging infectious diseases in high-income countries are also discussed, often implicating products imported across borders as well as trans-national spread due to as yet unknown causes. Other papers discuss zoonotic and vector-borne disease epidemiology and present opportunities for predictive modelling. Several diagnostic advances are also mentioned, elucidating changing epidemiological trends in recently recognised re-emerging pathogens.

This issue of *Epidemiology & Infection* represents a diverse overview of current concerns surrounding emerging infectious diseases globally. All highlight the importance of supporting ongoing surveillance efforts as the cornerstone of disease prevention. Early recognition of an outbreak allows control measures to be initiated in a timely way that can shift the epidemic curve, reducing its impact and possibly its geographic spread. Enhanced surveillance measures with an emphasis on innovation, transparency and incorporation of the One Health model are critical to epidemic preparedness measures in the future. It is also crucial to encourage research during outbreaks through rapid data sharing to facilitate rapid response efforts, as is promoted through organisations such as the International Severe Acute Respiratory and Emerging Infection Consortium (ISARIC) [9]. Vaccination, if it can be implemented in time, can also bend the epidemic curve. Promoting platforms for rapid vaccine development and deployment could provide a significant boost to outbreak control. The Coalition for Epidemic Preparedness Innovations (CEPI), a public–private coalition that has been working to halt epidemics through the development of appropriate vaccines, is promoting both pathogen-specific and agnostic platform approaches [10]. These efforts deserve wide support and encouragement. In order for vaccines to be effectively employed, the growing threat of vaccine hesitancy worldwide must also be countered using methods grounded in the social sciences. Governments, through their public health agencies and in coordination with efforts like the Global Health Security Agenda, must adopt preparedness plans and exercise them before outbreaks become major threats [11]. Emerging and re-emerging infectious diseases will continue to present significant challenges in the coming years, and investing in novel methods for detection, prevention as well as therapeutics should remain priorities for the global public health community.
